# Asymmetric effects of semantic compatibility and structural bridging on content propagation: evidence from community vernacular combinations on social media platforms

**DOI:** 10.1038/s41598-026-51438-6

**Published:** 2026-05-09

**Authors:** Jingjing Mu, Xing Yuan, Vasylieva Olena Serhiivna, Qian Zhao, Yue Zhu

**Affiliations:** 1 School of Art and design, Communication University of China, Nanjing, 211172 Jiangsu Province China; 2 Department of Multimedia Design, Kyiv National University of Technologies and Design, Kyiv, Ukraine; 3 School of Art & Design, Shaanxi University of Science & Technology, Xian, 710021 Shaanxi Province China; 4 School of Culture and Media, Hezhou University, Hezhou, 542899 Guangxi China; 5 Department of Art and Fashion Design, Kyiv National University of Technologies and Design, Kyiv, Ukraine; 6 Faculty of Fine and Applied Arts, Mahasarakham University, Maha Sarakham, Thailand

**Keywords:** Community vernacular, Semantic compatibility, Structural bridging, Platform propagation mechanism, Social network analysis, Complex networks, Complex networks, Mathematics and computing, Psychology, Psychology

## Abstract

The rapid expansion of social media platforms and the fragmentation of interest communities have created new theoretical demands for understanding content propagation. Users routinely combine community-specific semantic vernaculars in their content, yet combination-level propagation patterns remain invisible within single-vernacular frameworks. This study constructs a color vernacular co-occurrence network from ACG-related posts on Xiaohongshu (January 2024 to March 2025) and examines how cognitive hierarchy compatibility and network structural position jointly associate with combination propagation outcomes. Three findings emerge. First, semantic hierarchy compatibility positively associates with propagation synergy: within-level compatible combinations correspond to higher propagation gains and cross-level incompatible combinations correspond to negative synergy. Second, cognitive distance associates with propagation outcomes in a monotonically accelerating declining pattern, which does not support the inverted-U hypothesis, because the cognitive gains from semantic novelty cannot offset the decoding burden that accelerates as hierarchical distance increases. Third, semantic hierarchy and community structure show opposite directional associations: cross-level semantics correspond to a negative association while cross-community bridging corresponds to a positive association, and cognitively compatible cross-community combinations consistently outperform incompatible ones. Maintaining semantic hierarchy compatibility while pursuing structural bridging, and incorporating cognitive compatibility into platform recommendation mechanisms, are productive directions for future research.

**Supplementary Information:**

The online version contains supplementary material available at 10.1038/s41598-026-51438-6.

## Introduction

On social media platforms, the vocabulary used in post content and the propagation performance of that content are systematically associated. Word choice in post bodies influences how likely content is to be discovered by specific audiences through platform search, topic aggregation, and recommendation distribution, and differences in word usage exhibit observable regularities in relation to content visibility and user engagement^1,2^. Recent research on deep semantic modeling, recommendation systems, and multimodal analysis has shown that textual semantic features play an important role in platform content distribution^3–5^, and related methods have been widely applied to visibility prediction and user behavior analysis on social media^6–8^. Within various interest communities, user groups have widely developed community-specific semantic vernaculars, which they naturally embed in post bodies to mark content style and establish in-group identity. Such community vernaculars differ from topic hashtags explicitly appended with the “#” marker: hashtags are explicit, strategically manipulable metadata, whereas community vernaculars are platform-indexable semantic cues implicitly embedded in natural language, carrying both community identity marking and style framing functions, and are equally captured by platform search and recommendation systems, yet have long been overlooked within the traditional hashtag propagation research framework^9,10^.

When users combine multiple community vernaculars within the same post, the differences in propagation performance across different combinations far exceed what would be expected from the individual propagation capacities of each vernacular. Two combinations whose component vernaculars are comparable in individual propagation capacity may yield diametrically opposite actual propagation outcomes: some combinations perform significantly above the mean of their component vernaculars’ individual performances, while others systematically fall below this baseline. This non-additive pattern means that the intuitive logic by which content creators make combination decisions based on single-vernacular experience fails at the systemic level. The propagation patterns of vernacular combinations are invisible at the single-vernacular level and can only be observed and explained at the combination level. Existing research lacks explanations for this systemic pattern at the combination level, constraining deeper understanding of symbolic propagation mechanisms on platforms.

Cognitive psychology and network science each offer substantial explanatory power for the propagation effects of individual symbols. Cognitive psychology predicts that vernaculars with lower decoding thresholds obtain broader audience engagement due to higher cognitive accessibility^11,12^. Network science predicts that vernaculars occupying high-centrality or bridging positions obtain stronger diffusion capacity due to structural advantages^13,14^. However, both traditions were developed for single units of analysis and neither has developed boundary condition characterizations for vernacular pairs as the unit of analysis. Unlike prior integrative research^15,16^ that has primarily focused on single-node cognitive attributes, this study advances the unit of analysis to the node-pair level and positions cognitive compatibility as a moderating variable, systematically testing whether propagation gains from network structural bridging are systematically diminished when cognitive hierarchies are incompatible. Integrative research has been called for but remains in an early stage.

The central challenge in testing this proposition lies in the need for a community vernacular system whose cognitive hierarchies are clearly defined and operationalizable. However, the cognitive attribute boundaries of community vernaculars in most content domains are ambiguous, making systematic cognitive hierarchy classification difficult, with the result that the influence of cognitive hierarchy differences on combination effects has not yet been systematically testable. Color vernaculars in Animation, Comics, and Games (ACG)-related content on social media platforms offer unique research conditions for resolving this challenge. ACG color vernaculars exhibit naturally occurring tri-level naming mechanisms in their cognitive-cultural encoding: perceptual-level color vernaculars (e.g., “red”) correspond to basic hue categories with relatively lower cultural decoding thresholds [Basic color terms: Their universality and evolution.]; associative-level color vernaculars (e.g., “sakura pink”) point to specific hues through associations with natural or everyday scenes and require contextual experiential decoding; symbolic-level color vernaculars (e.g., “Hatsune green”) are bound to specific IP or subcultural identities and require in-group knowledge to understand [Reading images: The grammar of visual design]. This stratification is operationalized through replicable rules and validated through inter-annotator reliability testing, demonstrating that the classification can be reliably recognized. This complete natural variation in cognitive hierarchy from generic hue to proprietary symbol, which is difficult to replicate in other community vernacular systems, provides an ideal natural experimental setting for systematically testing how cognitive hierarchy differences affect combination propagation effects.

This study proposes a Dual Bridging Integration Framework, whose core claim is that cognitive compatibility is the key moderating variable affecting the magnitude of propagation gains from structural bridging. Community bridging at the network level produces independent positive propagation gains in combinations that are compatible at the cognitive hierarchy level. However, when a combination simultaneously spans cognitive hierarchies, the decoding burden generated by cognitive incompatibility systematically weakens the propagation advantage of structural bridging, substantially reducing bridging gains. This claim repositions the bridging effect from a purely structural mechanism to a joint structural-cognitive mechanism, providing an empirically testable boundary condition for the applicability of weak tie theory and structural hole theory in semantic symbol networks.

This study specifically addresses four questions: (1) Do color vernacular combinations produce synergistic propagation effects, and do different cognitive hierarchy combination types exhibit systematic differences? (2) What form does the association between paired vernaculars’ cognitive distance and propagation outcomes take, whether a linear decline or whether an optimal interval exists? (3) How does the centrality configuration of color vernacular pairs in the co-occurrence network associate with propagation outcomes, and is there a systematic difference in predictive power between degree centrality and PageRank? (4) Is the propagation gain from community bridging contingent on cognitive compatibility as a necessary precondition, and does bridging produce negative effects when cognitive incompatibility is present? The first two questions respectively confirm the main effect of the cognitive dimension and its directionality, the third confirms the main effect of the network dimension, and the fourth tests the threshold moderating role of cognitive compatibility on structural bridging. Together, the four questions constitute a progressive analytical logic from effect confirmation to boundary identification to mechanism integration.

### Literature review and theoretical framework

Understanding the propagation outcomes of community vernacular combinations requires integrating the cognitive decoding mechanisms of community vernaculars and their role patterns in network propagation. Cognitive processing theory emphasizes that different types of symbolic information exhibit systematic differences in decoding depth and required resources within the cognitive system^11^. The decoding complexity of color vernaculars is hierarchically differentiated along linguistic and cultural dimensions: vernaculars referring to basic hue categories can be understood without additional background knowledge; vernaculars named through contextual association require lived experience to activate the corresponding imagery^17^; vernaculars carrying cultural encoding require specific subcultural knowledge to complete decoding^18^. The cognitive complexity of color significantly influences attention allocation^19^, emotional arousal^20^, and memory retention^21^. In platform propagation contexts, the cognitive complexity of vernaculars affects content visibility and user engagement. Vernaculars of moderate complexity perform better in search rankings^22^, and vernaculars carrying cultural codes, though reaching a narrower audience, can stimulate strong resonance within in-groups^23^. Recent research on multimodal content analysis, knowledge graphs, and semantic representation learning has further shown that semantic hierarchy heterogeneity has differentiated effects on content distribution logic^24–27^. These empirical findings underscore the heterogeneity of color vernaculars across cognitive levels and their influence on platform propagation. However, perspectives focused on cognitive processing primarily attend to psychological mechanisms at the individual level while giving less consideration to how the structural position of color vernaculars in social networks affects their propagation capacity. In ACG content propagation on social media platforms, cognitive decoding mechanisms and network structural position are not independent: while the ease with which a color vernacular is decoded certainly affects its propagation potential, its position in the co-occurrence network equally determines the scale of audience it can reach. This limitation raises the question of understanding the role of color vernaculars’ network structural characteristics in platform propagation.

Research on color vernacular propagation requires shifting focus from individual cognition to network structural position. Network position theory holds that a node’s structural position in a network determines its information diffusion capacity^13,28^. Degree centrality measures a node’s direct connections; PageRank captures a node’s indirect influence through a recursive algorithm. Work applying network methods to color research and platform content propagation has revealed the influence of network structural characteristics on propagation. Color vernacular co-occurrence networks exhibit clear association patterns and community structures, with high-centrality color vernaculars demonstrating significant advantages in propagation^29^. In social media vernacular networks, high degree centrality vernaculars receive greater user adoption and content exposure^30,31^, and the PageRank values of vernaculars show positive correlations with the user engagement they generate^32^. However, when vernaculars appear in combination, research has found contradictory results. Some studies indicate that dual high-centrality vernacular combinations achieve the best propagation outcomes due to cumulative advantage^33^, but others find that combinations with one high and one low centrality vernacular actually perform better, with the low-centrality vernacular benefiting from a “leverage effect” from the high-centrality one^34^. These contradictory findings suggest that the propagation mechanism of vernacular combinations may be moderated by the semantic relationship between vernaculars, which is inherently a cognitive-dimensional variable. A purely network structural perspective cannot explain why structurally similar combinations exhibit markedly different propagation outcomes. Moreover, existing research has primarily focused on node centrality within a single network while giving less attention to how nodes expand propagation range by bridging different community boundaries. This limitation raises the necessity of understanding cross-community bridging in the propagation of color vernacular combinations.

Research on color vernacular combination propagation needs to be further elevated to the perspective of cross-community bridging. Bridging theory provides the theoretical foundation for understanding how spanning structural divides enhances information diffusion. Weak ties connect different circles that otherwise lack information exchange and hold unique advantages in information propagation^35^. Structural holes are gaps between two groups in a network that lack direct connections; nodes occupying this position can act as information brokers, gaining informational advantages and control benefits^14^. These theories have been validated in studies of platform propagation and cross-circle diffusion. On Twitter, content posted by users who bridge different communities receives broader retweets and cross-circle propagation^36^. In Instagram vernacular networks, vernacular combinations that bridge different interest communities receive more diverse audiences and higher engagement than within-community vernacular combinations^37^. However, research has also identified potential risks of bridging. When vernacular combinations span communities that are too dissimilar, content may face restricted propagation because it fails to align with the core expectations of either community^34^. These findings suggest that bridging effects may be moderated by the attribute compatibility between the bridged nodes. In the propagation context of color vernacular combinations, this attribute compatibility has a clear cognitive-dimensional correspondence: whether the cognitive decoding hierarchies between color vernaculars are consistent may determine whether bridging at the network level can be converted into actual propagation advantages.

Integrating the above three theoretical perspectives, this study constructs a Dual Bridging Integration Framework for understanding the propagation outcomes of color vernacular combinations. “Dual bridging” refers to bridging in the cognitive dimension and bridging in the network dimension: the former occurs when color vernaculars with different cognitive decoding hierarchies are used in combination, and the latter occurs when color vernaculars belonging to different communities are used in combination. In contrast to a parallel hypothesis that treats the two types of bridging as independent mechanisms, and unlike prior integrative research^15^ that has primarily focused on single-node cognitive attributes, the core claim of this framework is that cognitive compatibility constitutes the key moderating variable affecting the magnitude of propagation gains from community bridging at the network level. When a color vernacular combination maintains compatibility at the cognitive hierarchy level, community bridging at the network level can produce independent propagation gains. Whereas when a combination simultaneously spans cognitive hierarchies and community boundaries, the decoding burden generated by cognitive incompatibility systematically weakens the propagation advantage of structural bridging, substantially reducing bridging gains. This claim repositions the bridging effect from a single structural mechanism to a joint structural-cognitive mechanism, providing testable empirical boundary conditions for the applicable scope of weak tie theory and structural hole theory in semantic symbol networks. This study uses the naturally occurring three-tier cognitive hierarchy structure of ACG color vernaculars as the testing context to systematically examine how the cognitive hierarchy characteristics and network structural positions of vernacular combinations jointly associate with their platform propagation performance.

## Methodology

### Data source and sample

The data for this study are from the Xiaohongshu platform. Xiaohongshu is China’s leading image-text content community platform, which brings together a large number of Generation Z ACG content creators and consumers and is one of the primary venues for high-frequency use and natural propagation of ACG color vernaculars^38^. The data collection timeframe spans January 2024 to March 2025; image-text posts annotated with ACG color vernaculars were systematically retrieved using web crawler tools, with each record containing the post body, color vernacular annotations, comment count, like count, and share count. The following processing steps were applied to the sample and data to satisfy the requirements of the research: (1) posts containing explicit ACG color vernacular annotations in the body text were retained; (2) based on the number of color vernaculars in each post, the sample was divided into multi-color posts (containing 2 or more color vernaculars) and single-color posts (containing only 1 color vernacular), with the former constituting the primary analytical sample for combination propagation analysis and the latter serving as the single-color baseline sample for synergy effect calculation; (3) samples with missing values on key variables were excluded; (4) to preserve as many valid samples as possible, samples with missing baselines for synergy effect calculation were not deleted at the primary analysis stage but are separately reported through robustness subset analysis. After the above processing, this study obtained a final set of 38,564 valid posts, comprising 8,620 multi-color posts (22.35%) and 7,840 single-color posts (20.33%); the two types of posts show no systematic differences in collection timeframe, topic distribution, or user characteristics, satisfying the comparability requirements for baseline comparison.

Color vernacular information was extracted from post text rather than relying on image pixel analysis, because it is the color vocabulary explicitly annotated by users in post bodies that affects platform content visibility and recommendation distribution. The extraction process employed the RoBERTa-wwm-ext pretrained model with domain-adaptive fine-tuning^39,40^, to enhance the model’s recognition of ACG domain-specific color vocabulary. On a manually annotated test set (*n* = 5,000 posts), overall precision was 0.89, recall 0.85, and F1 score 0.87. Per-class evaluation results are reported in Table NER: perceptual-level color vernaculars (basic hue words) achieved the highest recognition accuracy (F1 = 0.91), symbolic-level vocabulary achieved relatively lower accuracy (F1 = 0.67) due to limited training samples, and associative-level vernaculars fell between the two (F1 = 0.83). This is consistent with the objective constraint that rare cultural symbol vocabulary has limited available training samples.

**Table 1:** Per-class NER classifier performance (manually annotated test set, *n* = 5,000).

Class	Precision	Recall	F1	Support
Perceptual (basic hue words)	0.92	0.90	0.91	3,200
Associative (contextual words)	0.87	0.80	0.83	1,560
Symbolic (IP/subculture words)	0.68	0.65	0.67	240
Weighted average	0.89	0.85	0.87	5,000

Perceptual-level precision is highest, reflecting the wide distribution of basic hue words in the corpus. Symbolic-level precision is lowest, consistent with limited training samples for IP-bound vocabulary. The weighted average is consistent with the overall metrics reported in the text.

To assess the impact of classification errors on the primary conclusions, supplementary analyses simulated reclassification noise rates of 2% to 10% for both is_cross_layer and cog_distance (50 repetitions each). At all noise rates, the OLS significance rate was 100% and the directional accuracy rate was 100% (Supplementary Table S3), indicating that the conclusions are highly robust to NER classification errors^41^. All color vernacular pair combinations were extracted from posts containing multiple color vernaculars and deduplicated to form the analytical sample.

### Variable definitions

The composite propagation outcome index (P) is the dependent variable, quantifying the degree of user engagement activity that each post generates on the platform. This study employs Principal Component Analysis (PCA) to objectively determine the weights of three indicators, namely comment count, like count, and share count, to avoid the circular measurement problem that arises from using the distribution of the dependent variable itself to determine weights. The three engagement indicators for multi-color posts were log-transformed and standardized separately; the purpose of log-transformation is to eliminate the positive skewness of engagement indicators so that PCA extracts the first principal component based on a more symmetric variance structure. The first principal component was extracted with a variance explained rate of 80.7%, indicating that the three indicators highly jointly reflect the underlying construct of “propagation activity.” The absolute values of the first principal component loadings were normalized as weights, yielding a comment weight of 0.320, like weight of 0.350, and share weight of 0.330. The composite propagation outcome index is calculated as:

1 $$\:P=0.320\:\cdot\:{{ln}\left(C+1\right)}_{z}+0.350\:\cdot\:{{ln}\left(L+1\right)}_{z}+0.330\:\cdot\:{{ln}\left(S+1\right)}_{z}$$

where *C*,* L*, and *S* are comment count, like count, and share count respectively, and the subscript *z* denotes standardization. The entropy weighting method and equal weighting were employed as comparative schemes to verify robustness; the three schemes yield highly consistent results, indicating that weight selection has no material effect on the core conclusions.

The core unit of analysis is the color vernacular pair, defined as the unique combination of two color vernaculars within the same post. All pairwise combination instance-level records were extracted from multi-color posts and aggregated by unique color pair; the propagation outcome for each color pair is the mean P value across all co-occurring posts for that combination. Descriptive statistics for the resulting analytical sample are reported in Table 1. All statistical tests are conducted on aggregated data at the unique color pair level rather than on instance-level data, to avoid the pseudo-replication problem arising from multiple color pairs within the same post sharing the same propagation value. To verify the validity of pair-level aggregated inference, supplementary analyses estimated OLS regression at the instance level using color pair as the clustering unit (pair-clustered robust standard errors); results are reported in Supplementary Table S1 and are directionally consistent with the pair-level primary analysis, confirming that pair-level aggregation does not introduce inferential bias.

This study constructs independent variables along two dimensions: cognitive and network. On the cognitive dimension, based on the cognitive hierarchy tier to which each member of a color pair belongs, three indicators are constructed: combination type (three within-level and three cross-level types, six in total), cognitive hierarchy distance (within-level = 0, adjacent-level = 1, across-two-levels = 2), and a binary variable for whether the pair crosses cognitive levels, together characterizing the degree of compatibility between color pairs in the cognitive-semantic dimension. On the network dimension, the PageRank centrality, degree centrality, and betweenness centrality of each node are extracted from the color vernacular co-occurrence network; network characteristics at the color pair level are taken as the mean of the corresponding metrics for the two endpoint nodes; community relationship is classified into within-community combinations and cross-community combinations based on whether the two endpoint nodes belong to the same Louvain community.

Synergy effect, as the core indicator measuring combination propagation gain, is defined as the difference between the actual propagation outcome of a color pair and its expected baseline value from single-color posts. Let *E*_*i*_ and *E*_*j*_ be the mean propagation outcomes of color vernaculars *i* and j in single-color posts respectively; the synergy effect is calculated as:

2 $$\:{\mathrm{Synergy}}_{ij}={\stackrel{-}{P}}_{ij}-\frac{{E}_{i}+\:{E}_{j}}{2}$$

The baseline here uses the arithmetic mean (*E*_*i*_ + *E*_*j*_)/2, reflecting an additive null hypothesis of “zero interaction,” treating the two vernaculars as independently contributing to propagation; deviations from this baseline constitute the synergy effect. This additive formulation is consistent with standard practice in information combination research and theoretically corresponds to a null hypothesis of fully additive decomposition of propagation outcomes. As a robustness check, OLS regression-predicted values based on each vernacular’s individual attributes were simultaneously employed as an alternative baseline, yielding directionally consistent results with the primary analysis. The single-color baseline comes from the mean propagation outcomes of each color vernacular when appearing alone in single-color posts, to ensure consistency in measurement calibration between the baseline and the combination context. Synergy effect calculation requires that both color vernaculars appear at least 5 times in single-color posts; color pairs not meeting this condition are concentrated primarily among associative-level and symbolic-level vocabulary, reflecting the objective constraint that rare cultural symbol vocabulary lacks sufficient single-color reference samples. This study reports full-sample analysis and reliable-baseline subset analysis in parallel to assess the impact of this limitation on conclusion robustness.

Additionally, the log values of the co-occurrence frequency and instance count of color pairs are used as control variables, to control for the potential sample size stability advantage that high-frequency combinations may have in propagation outcome measurement.

### Analytical strategy

The analytical strategy of this paper proceeds in four progressive levels.

First, synergy effect testing. One-sample t-tests are used to test whether the mean synergy effect for each combination type deviates significantly from zero, to determine whether there is a systematic difference between the actual propagation performance of color vernacular combinations and the expected single-color baseline value. Welch t-tests are used to compare mean synergy effects between within-level and cross-level combinations, to examine the pattern of association between cognitive hierarchy consistency and synergy effects. All tests are conducted on aggregated data at the unique color pair level, with effect sizes reported as Cohen’s d. Each test addresses an independent research question; Bonferroni correction was not applied, and effect sizes are reported throughout to support magnitude-based interpretation.

Second, testing the functional relationship between cognitive distance and propagation outcomes. To test whether the relationship between cognitive hierarchy distance and propagation outcomes is linear or exhibits an optimal interval, the Jonckheere-Terpstra (*JT*) trend test is first employed as the primary criterion for monotone trend, directly testing whether the three group means of propagation outcomes (d = 0, d = 1, d = 2) follow a strictly monotone decreasing trend. Bootstrap accelerated contrast testing (10,000 iterations) is used as the criterion for declining acceleration, by comparing whether the difference between the between-group declines delta_01 and delta_12 is significantly greater than zero. As auxiliary analysis, an OLS regression model is estimated simultaneously including linear and quadratic terms for cognitive distance, specified as:

3 $$\:{\stackrel{-}{P}}_{ij}=\alpha\:+{\beta\:}_{1}{d}_{ij}+{{\beta\:}_{2}d}_{ij}^{2}+{\beta\:}_{3}{ln}\left({n}_{ij}\right)+{\epsilon}_{ij}$$

where *d*_*ij*_ is the cognitive hierarchy distance of color pair (*i*,* j*), *n*_*ij*_ is the number of co-occurrence instances, and all regressions use HC3 heteroscedasticity-robust standard errors. Since cognitive distance contains only three discrete values (0/1/2), fitting a quadratic curve to three points has inherent statistical limitations; the quadratic regression serves only as an exploratory auxiliary tool for describing the direction of accelerating decline. Primary conclusions rest on the JT trend test and Bootstrap accelerated contrast test results. Model A, using a binary variable for whether the pair crosses cognitive levels in place of the continuous distance, serves as the baseline model; all continuous variables are standardized.

Third, testing network embedding effects. Color pairs with a co-occurrence frequency no less than 5 times are retained using a pre-specified threshold to ensure sufficient statistical stability; this threshold was determined a priori based on the criterion that estimation standard errors are too large for low-frequency color pairs, rather than being selected post hoc based on analytical results. Pearson and Spearman rank correlations between each network centrality indicator and color pair propagation outcomes are computed; prior to regression analysis, all centrality indicators are checked for variance inflation factor (VIF), and highly collinear variables with VIF greater than 10 are excluded. To increase analytical transparency, complete sensitivity analysis results for co-occurrence frequency thresholds of 1, 3, 5, 10, and 20 are simultaneously reported.

Fourth, testing community bridging effects and moderation patterns. Welch t-tests are used to compare propagation outcome differences between cross-community and within-community combinations, to examine the direction and strength of association between community boundary spanning and propagation performance. Building on this, a 2 × 2 cross-tabulation matrix of cognitive hierarchy and community relationship is constructed to describe the joint distribution patterns of the two dimensions, identifying the moderating pattern of cognitive compatibility on community bridging propagation performance. The following OLS regression model is further estimated to independently quantify the association strength of each dimension with propagation outcomes while controlling for other variables:

4 $$\:{\stackrel{-}{P}}_{ij}=\alpha\:+{\beta\:}_{1}{\mathrm{Cross}}_{ij}+{\beta\:}_{2}{\mathrm{Bridge}}_{ij}+{\beta\:}_{3}{\mathrm{Degree}}_{ij}+{\beta\:}_{4}{ln}\left({n}_{ij}\right)+{\epsilon}_{ij}$$

where *Cross*_*ij*_ is the binary variable for whether the pair crosses cognitive levels, *Bridge*_*ij*_ is the binary variable for whether the pair crosses community boundaries, and Degree_ij is the mean degree centrality of the color pair. The directions and relative magnitudes of coefficients *β₁* and *β₂* are compared to contrast the differences and similarities in the associative effects of cognitive hierarchy and community relationship on propagation outcomes. All models use HC3 heteroscedasticity-robust standard errors, and 95% confidence intervals are calculated as beta plus or minus 1.96 times SE^42^.

## Results

### Synergistic propagation effects of color vernacular combinations

This section focuses on whether color vernacular combinations produce synergistic propagation effects and whether there are systematic differences among different cognitive hierarchy combination types. Table 2 reports descriptive statistics for all analytical variables, and Table 3 reports synergy effect testing results for the six combination types.

One-sample *t*-tests conducted on 3,108 unique color pairs with reliable single-color baselines show that the mean synergy effect is 0.398, significantly deviating from zero (*t* = 22.53, *p* < 0.001), with a positive synergy proportion of 63.03%. Among the six combination types, within-level perceptual-level combinations and within-level associative-level combinations both show significantly positive synergy effects, while the two combination types spanning the greatest cognitive distance (perceptual-level to symbolic-level cross-level and associative-level to symbolic-level cross-level combinations) show significantly negative synergy effects (Table 3). Classifying all color pairs into within-level and cross-level categories, the Welch *t*-test shows that the mean synergy effect for within-level combinations (*M* = 0.461) is significantly higher than for cross-level combinations (*M* = 0.330; *t* = − 3.706, *p* < 0.001, *d* = − 0.133). Repeating the above analysis on the subset with reliable baselines yields conclusions consistent in direction.

**Table 2:** Descriptive statistics of variables.

Variable	*N*	Mean	SD
Mean propagation outcome	21,105	0.721	1.113
Synergy effect	3,108	0.398	0.984
Cognitive hierarchy distance	21,105	0.832	0.561
Whether cross-level (binary)	21,105	0.524	0.499
Mean degree centrality	824	0.277	0.098
Mean PageRank centrality	824	0.035	0.018
Whether cross-community (binary)	824	0.625	0.484
Co-occurrence frequency	21,105	12.41	38.76

**Table 3:** Synergy effect testing results for six combination types.

Combination type	*n*	Mean synergy	SD	95% CI	t	*p*	Cohen’s d	Positive synergy (%)
Within-level perceptual	1,308	0.459	0.904	[0.410, 0.508]	18.37	< 0.001	0.508	69.65
Within-level associative	309	0.469	1.177	[0.338, 0.600]	7.00	< 0.001	0.398	59.55
Perceptual–associative cross-level	1,433	0.348	1.020	[0.295, 0.401]	12.92	< 0.001	0.341	59.04
Perceptual–symbolic cross-level	36	−0.116	0.177	[− 0.174, − 0.058]	−3.91	< 0.001	−0.652	27.78
Associative–symbolic cross-level	21	−0.161	0.265	[− 0.274, − 0.048]	−2.78	0.012	−0.607	33.33
Within-level symbolic	1	-	-		-	-	-	-

Note: One-sample t-test (H₀: mean synergy = 0). 95% CI = mean ± 1.96 × SE. Within-level symbolic combinations are excluded from testing due to insufficient sample size.

### Association pattern between cognitive distance and propagation outcomes

**Fig. 1:**
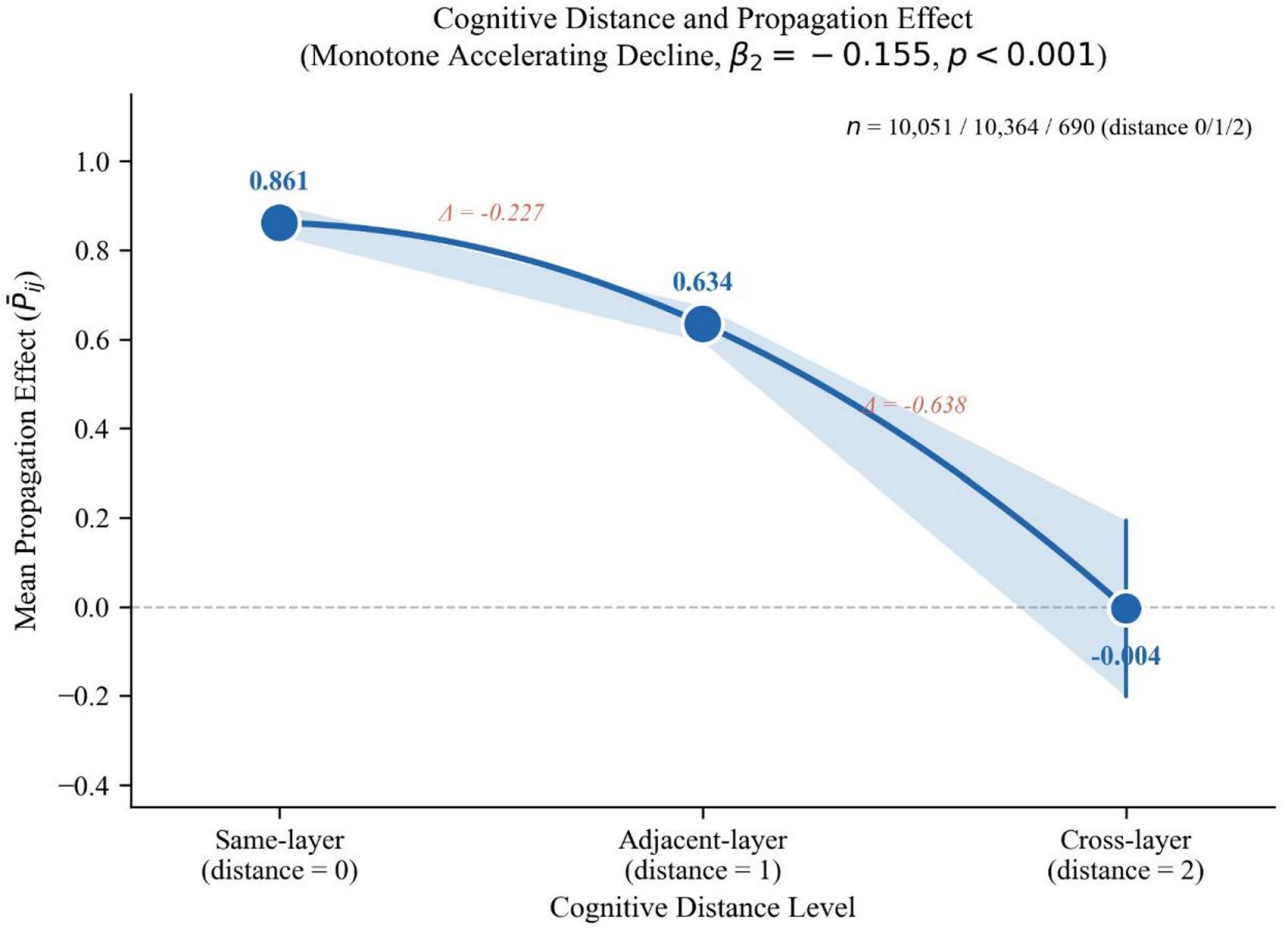
Cognitive distance and propagation effect: evidence for monotone accelerating decline. Note: The x-axis represents three cognitive distance levels: d = 0 (within-level, n = 10,051), d = 1 (adjacent-level, n = 10,364), and d = 2 (across-two-levels, n = 690). Points show group means; the shaded band shows 95% confidence intervals. Annotated Δ values indicate between-group declines. The fitted curve is a quadratic spline for visual guidance only. Primary evidence for monotone accelerating decline rests on the Jonckheere-Terpstra trend test (z = 20.008, p < 0.001) and Bootstrap accelerated contrast test (Δ₁₂ − Δ₀₁ = 0.410, 95% Bootstrap CI [0.342, 0.480], p < 0.001); the quadratic term (β₂ = −0.155, p < 0.001) is auxiliary.

This section tests the functional form of the relationship between cognitive hierarchy distance and propagation outcomes. Figure 1 presents the mean propagation outcomes and 95% confidence intervals for the three cognitive distance groups; propagation outcomes decline monotonically as cognitive distance increases (within-level: *M* = 0.861, 95% *CI* [0.840, 0.883]; adjacent-level: *M* = 0.634, 95% *CI* [0.611, 0.656]; across-two-levels: *M* = −0.004, 95% *CI* [−0.052, 0.045]), with ANOVA results significant across the three groups (*F* = 255.71, *p* < 0.001, eta^2 = 0.024). The JT trend test confirmed a significant monotone decreasing trend, and Bootstrap accelerated contrast testing showed that the between-group decline accelerates at higher cognitive distances, supporting the pattern of accelerating decline (Table 4).

The *d* = 2 group (across two cognitive levels) is structurally composed entirely of perceptual-to-symbolic and associative-to-symbolic cross-level combinations that include symbolic-level vernaculars. In the symbolic-layer exclusion sensitivity analysis (Supplementary Table S4), removing symbolic-layer pairs causes the *d* = 2 group to become empty, while the monotone decline between the perceptual and associative layers (d = 0 vs. *d* = 1) remains significant (*M* = 0.889 vs. 0.648, *p* < 0.001), indicating that the perceptual-associative trend does not depend on symbolic-layer pairs.

**Table 4:** OLS regression results: association pattern between cognitive distance and propagation outcomes.

Variable	Model A (Binary Cross-Level)			Model B (Continuous Distance + Quadratic)	
	β	SE_HC3_	95% CI	β	SE_HC3_
Intercept	0.870***	0.011	[0.849, 0.892]	0.721***	0.008
Cross-level (binary)	−0.284***	0.015	[−0.313, −0.255]	-	-
Cognitive distance (linear)	-	-		−0.027	0.017
Cognitive distance (quadratic)	-	-		−0.155***	0.014
Instance count (log, standardized)	−0.146***	0.005	[−0.156, −0.136]	−0.145***	0.005
Adjusted R²	0.030	-		0.040	-
N	21,105	-		21,105	-
JT trend test	**z = 20.008**, *p < 0.001*			-	
Bootstrap acceleration test	**Δ₁₂−Δ₀₁ = 0.410**,** 95% CI [0.342**,** 0.480]**,*p* < 0.001			-	

Note: *** *p* < 0.001; HC3 heteroscedasticity-robust standard errors; all continuous variables standardized. Model B is exploratory auxiliary description; since cognitive distance contains only three discrete values (0/1/2), primary conclusions are based on the Jonckheere-Terpstra trend test (z = 20.008, *p* < 0.001) and the Bootstrap accelerated contrast test (acceleration magnitude = 0.410, 95% CI [0.342, 0.480], *p* < 0.001).

### Association between network embedding position and propagation outcomes

**Fig. 2:**
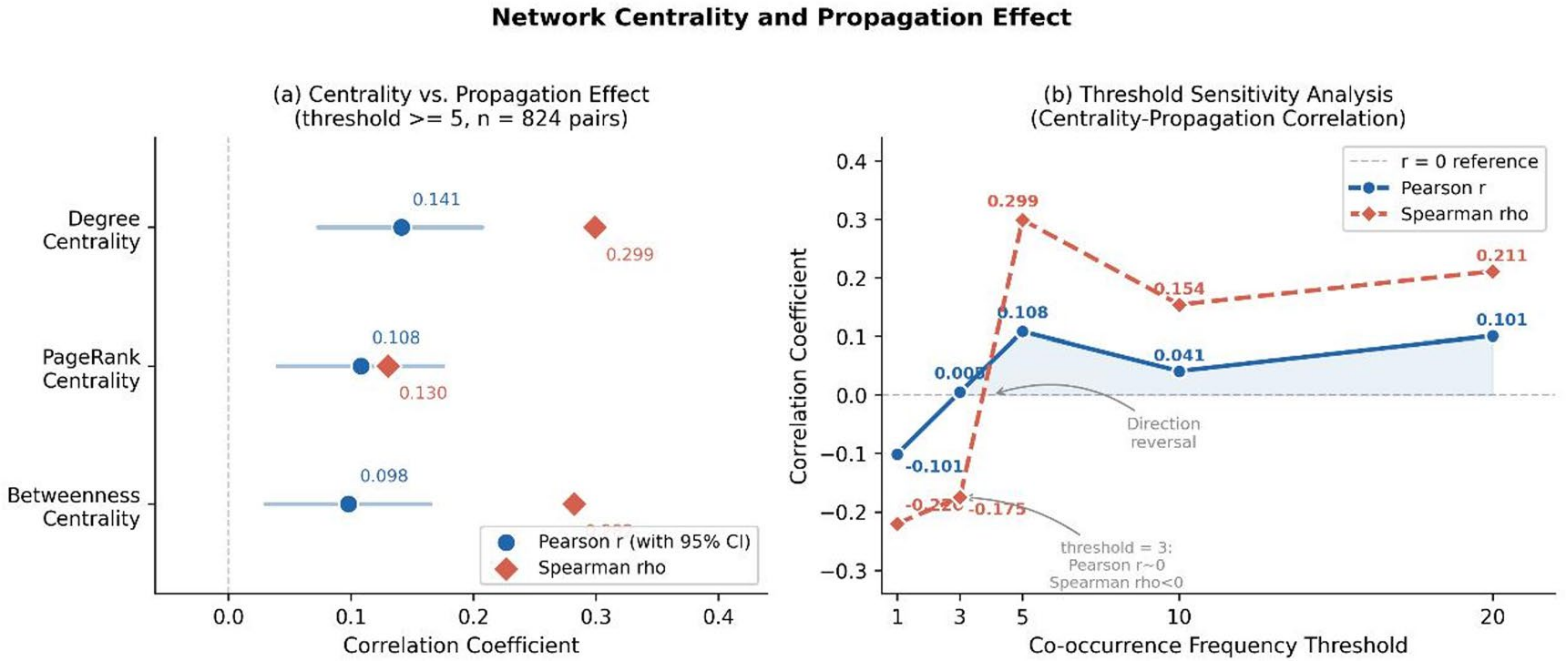
Network centrality and propagation effect: correlation estimates and threshold sensitivity analysis.

This section examines the association between the centrality positions of color vernaculars in the co-occurrence network and propagation outcomes. Using a pre-specified threshold of co-occurrence frequency no less than 5, 824 color pairs with sufficient statistical stability are retained. Degree centrality (Pearson *r* = 0.141, 95% *CI* [0.073, 0.207]), PageRank centrality (*r* = 0.108, 95% *CI* [0.040, 0.175]), and betweenness centrality (*r* = 0.098, 95% *CI* [0.030, 0.165]) all show significant positive associations with propagation outcomes; Spearman rank correlations are directionally consistent with Pearson correlations, with degree centrality yielding the highest Spearman ρ = 0.299, PageRank centrality ρ = 0.130, and betweenness centrality ρ = 0.282 (Fig. 2a).

At a threshold of 1 (full sample of 21,105 pairs), the Pearson correlation coefficient is − 0.101, indicating a negative association; at a threshold of 3, both Pearson r and Spearman ρ remain negative or near zero; at a threshold of 5 (824 pairs), the correlation turns positive (*r* = 0.108); at thresholds of 10 and 20, the positive association direction remains stable (Fig. 2b). This pattern indicates that there is a systematic difference in the direction of association between centrality and propagation outcomes for low-frequency versus high-frequency color pairs.

### Community bridging effects and the joint association with cognitive compatibility

This section examines the association between community boundary spanning and propagation outcomes, and the moderating role of cognitive hierarchy compatibility on this association pattern. Among the 824 color pairs meeting the frequency threshold, cross-community combinations (*M* = 0.159) show significantly higher mean propagation outcomes than within-community combinations (*M* = − 0.003; *t* = 5.213, *p* < 0.001, *d* = 0.376).

**Fig. 3:**
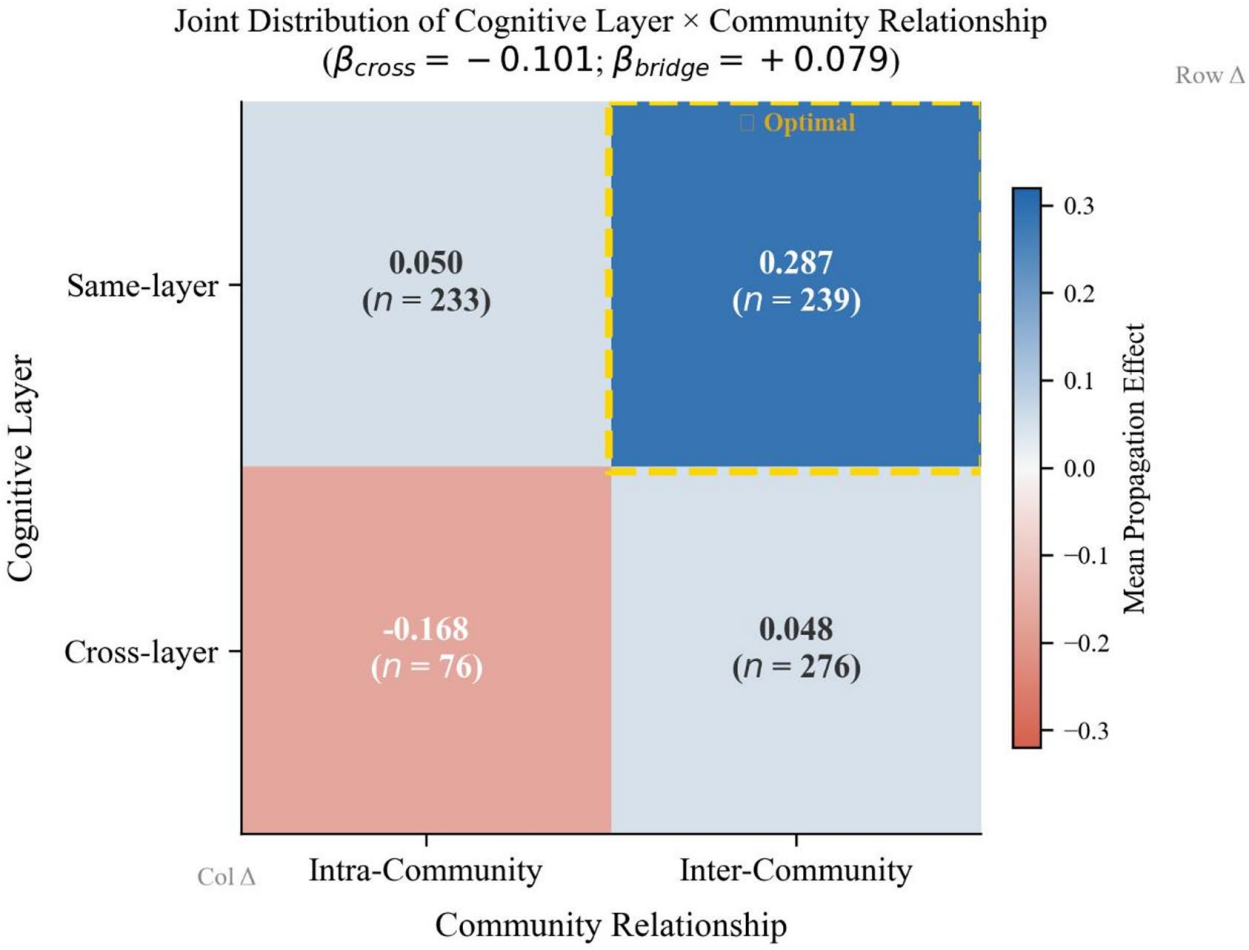
Joint distribution of cognitive layer compatibility and community structure on propagation effect. Note: Rows indicate cognitive hierarchy (same-layer vs. cross-layer); columns indicate community structure (intra-community vs. inter-community). Cell values show mean propagation outcome P̄_i_ⱼ with sample size n. Color intensity reflects propagation magnitude (blue: positive; red: negative). Row Δ and Col Δ show marginal differences. The dashed gold border marks the highest-performing quadrant. OLS regression (*n* = 824 pairs; co-occurrence frequency ≥ 5): β_cross-level = − 0.101 (SE = 0.016, *p* < 0.001); β_inter-community = + 0.079 (SE = 0.019, *p* < 0.001); HC3 standard errors.

Figure 3 presents the joint distribution patterns of cognitive hierarchy and community relationship. Within the four quadrants, within-level cross-community combinations yield the highest propagation outcomes (*M* = 0.287), while cross-level within-community combinations yield the lowest (*M* = − 0.168); cross-level cross-community combinations (*M* = 0.048), though higher than cross-level within-community combinations, still show a substantial gap compared to within-level cross-community combinations. OLS regression results show that, controlling for degree centrality and co-occurrence frequency, the coefficient for whether the pair crosses cognitive levels is significantly negative and the coefficient for whether the pair crosses community boundaries is significantly positive (Table 5). The two dimensions’ associations are in opposite directions. An alternative model substituting PageRank for degree centrality yields directionally consistent results.

**Table 5:** Joint analysis of community bridging effects and cognitive compatibility.

OLS Regression Coefficients: Independent Association Estimates for Propagation Outcomes								
Variable	Model A (β)	SE_HC3	*p*	95% CI	Model B (PageRank)	SE_HC3	*p*	95% CI
Cross-level (binary)	−0.101	0.016	< 0.001	[−0.133, −0.069]	−0.107	0.016	< 0.001	[−0.138, −0.076]
Cross-community (binary)	+ 0.079	0.019	< 0.001	[0.042, 0.117]	+ 0.099	0.019	< 0.001	[0.061, 0.136]
Mean centrality	+ 0.091 (degree)	0.019	< 0.001	[0.054, 0.129]	+ 0.052(PageRank)	0.020	0.010	[0.013, 0.091]
Co-occurrence frequency	−0.025	0.013	0.059	[−0.051, 0.001]	−0.000	0.015	0.989	[−0.029, 0.029]
R^2^	0.124	-			0.108	-		
N	824	-			824			

Beta values are X-standardized OLS coefficients with Y unstandardized; HC3 heteroscedasticity-robust standard errors. *** *p* < 0.001, ** *p* < 0.01, * *p* < 0.05, n.s. p > = 0.05. Model A is recommended.

## Discussion

### Core findings

First, this study examined the pattern of association between semantic hierarchy compatibility and combination propagation synergy effects. The results indicate a systematic positive association between semantic hierarchy compatibility and combination propagation synergy effects; within-level cognitive compatibility corresponds to higher combination propagation gains, and cross-level cognitive incompatibility corresponds to negative synergy effects. This result is directionally consistent with discussions in cognitive load theory: a shared decoding framework helps reduce the information processing burden on audiences, thereby corresponding to higher engagement willingness^43^. It also aligns with the findings of Heckler and Childers^44^, who suggested that semantic incongruity systematically reduces information acceptance. Compared to prior studies focusing on the propagation capacity of single nodes^45^, this study reveals a systematic association pattern between semantic compatibility and synergistic propagation performance at the node-pair level. Building on this result, the study further examines the functional form of the relationship between cognitive distance and propagation outcomes, to clarify how the theoretical divergence between the optimal interval hypothesis and the monotone decline hypothesis in existing literature manifests in social media contexts.

Second, this study examined the functional form of the relationship between cognitive distance and propagation outcomes. Prior research has provided relatively substantial literature support for the inverted-U optimal interval hypothesis in contexts of aesthetic evaluation and advertising acceptance, arguing that moderate semantic novelty can form a positive balance between cognitive burden costs and novelty gains^46,47^. In the present study, conducted in the context of social media color vernacular combination propagation, the observed association pattern is one of monotonically accelerating decline, which does not support the inverted-U hypothesis and is more consistent with the monotone negative relationship found by Heckler and Childers^44^ and Unnava and Burnkrant^48^ in information processing contexts. One explanation that is internally consistent with cognitive load theory for this contextual difference is that the time devoted to consuming social media content is extremely brief; audiences in rapid-browsing contexts are unable to fully realize the cognitive gains from semantic novelty, while cognitive burden accumulates at an accelerating rate with increasing hierarchical distance. This makes the precondition for the moderate-zone positive effect assumed by the inverted-U untenable in this context. Because this study’s data are cross-sectional observations, the above explanation provides a theoretically coherent framework for the cross-sectional association rather than a direct verification of causal mechanisms. Having confirmed the association pattern in the cognitive dimension, whether the network structural dimension can provide independent explanatory power for propagation, and how the effects of the two dimensions jointly manifest, constitute questions requiring further answers.

Finally, this study examined the association between network embedding position and propagation outcomes, and the joint association pattern of the semantic dimension and structural dimension with propagation outcomes. Network science and platform communication research both treat structural position as an important condition affecting content diffusion capacity^35,49,50^, with high-centrality nodes and bridging-position nodes expected to have propagation advantages due to broader reach. The positive association between centrality and propagation outcomes observed in high-frequency stable node pairs aligns with existing theoretical frameworks. The systematic directional shift observed among low-frequency node pairs can be understood from the following perspective: low-frequency color pairs have limited exposure opportunities on the platform due to rare usage, and low-frequency vocabulary may generate scarcity signal effects^51^; meanwhile, high-centrality vocabulary (such as basic hue words) is used so frequently that it may produce information saturation effects, reducing its marginal contribution to propagation^52^. This suggests that the propagation implications of centrality in platform communication contexts are modulated by node usage frequency, leading to differentiated association patterns^53,54^. At the level of joint analysis of the semantic and structural dimensions, the positive association between community boundary spanning and propagation outcomes is consistent with the core arguments of weak tie theory and structural hole theory. When both dimensions are incorporated into the analytical framework simultaneously, cross-level semantics correspond to a negative association while structural cross-community spanning corresponds to a positive association; the two exhibit a systematically asymmetric and opposing pattern. The mean propagation outcome of cross-level cross-community combinations (M = 0.048) is positive but substantially lower than that of within-level cross-community combinations (M = 0.287); the difference of 0.239 constitutes quantitative evidence of the cognitive compatibility moderating effect. The structural bridging advantage in reaching broader audiences can partially offset the cognitive decoding burden, but when both cognitive incompatibility and structural bridging are present simultaneously, this offset is insufficient. This asymmetric pattern has not been directly reported in existing integrative research, and is directionally consistent with the theoretical lineage of Centola and Macy^55,56^ regarding the constraining effect of cognitive processing demands on structural propagation conditions. These findings indicate that in the context of platform semantic symbol propagation, the semantic dimension and structural dimension jointly explain propagation outcomes through independent associations of opposite directions, providing a new analytical perspective for platform content propagation mechanism research and laying the foundation for future examination of the applicability of the dual-dimensional integration framework in other platform contexts.

### Theoretical contributions and practical recommendations

The rapid expansion of social media platforms and the deepening fragmentation of content communities have generated new theoretical demands for understanding platform propagation mechanisms. As various interest communities continue to develop proprietary semantic vernacular systems on platforms, the cognitive and structural logic that content creators follow when combining these vernaculars has an increasingly significant influence on propagation performance. In the context of the Xiaohongshu ACG color vernacular network, this study systematically examined the joint association pattern of semantic hierarchy compatibility and network structural bridging on propagation outcomes, and revealed an asymmetric pattern in which the two dimensions associate in opposite directions, as well as the finding that the cognitive distance effect follows a monotonically accelerating declining functional form rather than an inverted-U in social media contexts. Unlike research approaches based on graph neural networks and structural semantic modeling^57–59^, this study focuses on explaining observational propagation regularities from cognitive mechanisms and social network theory; the two perspectives are complementary: computational methods are strong at prediction and feature extraction, while this study’s framework provides interpretable mechanistic boundary conditions. These findings are not confined to a specific content type or platform context and hold theoretical reference value for research on platform content propagation in other communities with stratified vernacular systems. Grounded in objective data from the Xiaohongshu platform, this study provides a replicable empirical test case for a dual-dimensional propagation framework integrating cognitive processing theory and social network theory, not only pointing to the direction of theoretical deepening for domestic platform content propagation research, but also providing empirical reference from a Chinese social media platform for the international communication scholarship’s pursuit of integrative research in semantic symbol networks and platform algorithm contexts.

The following practical recommendations can be drawn from the findings of this study. First, when combining community vernaculars, content creators should treat semantic hierarchy compatibility as the priority consideration dimension of combination strategy. In this study’s context, within-level semantically compatible combinations systematically associate with higher combination propagation synergy effects, while cognitively hierarchy-incompatible combinations correspond to negative synergy, suggesting that maintaining consistency in cognitive decoding levels in vernacular selection is associated with more stable propagation gains. Second, when designing content recommendation and topic aggregation mechanisms, platform operators may incorporate the semantic hierarchy compatibility between vernacular pairs as a reference dimension in content quality evaluation, rather than relying solely on the historical propagation capacity of individual vernaculars for prediction. Third, when pursuing cross-circle propagation coverage, the coordination of structural bridging with semantic compatibility carries important reference value. This study finds that the positive association of semantically hierarchy-compatible cross-community combinations with propagation outcomes is significantly stronger than that of semantically hierarchy-incompatible cross-community combinations, suggesting that seeking structural bridging under the precondition of maintaining cognitive decoding level compatibility, compared to a strategy that pursues only network cross-boundary spanning while neglecting semantic compatibility, corresponds to higher mean propagation outcomes. Fourth, when using network centrality indicators to predict propagation performance, content researchers should clearly report sample frequency screening thresholds and conduct sensitivity analyses, because this study shows that the direction of the association between centrality and propagation outcomes changes systematically with threshold, and ignoring this dependency may lead to directional bias in conclusions about the propagation advantages of centrality. Fifth, community vernacular systems with clearly defined cognitive hierarchy classifications can serve as ideal contexts for operationalizing semantic-dimensional variables in platform propagation mechanism research; researchers may refer to the analytical framework of this study to advance comparable empirical research in other content communities with similarly stratified attributes^60^.

### Practical implications, limitations, and future work

This paper also has several limitations, from which the following directions for future research are proposed. First, constrained by data collection conditions, this study covers only post data from the Xiaohongshu platform within the specific time window of January 2024 to March 2025, and cannot fully reflect the propagation patterns of ACG color vernaculars over longer time spans or across other Chinese social media platforms. Moreover, the composite propagation outcome index in this study incorporates only three engagement indicators, namely comments, likes, and shares, without being able to include other dimensions such as read count and collection count; community structure was also detected by the Louvain algorithm on a co-occurrence network from a single cross-sectional timepoint, making the results subject to some algorithmic dependence. Both the measurement dimensions of propagation outcomes and the method of community delineation are amenable to refinement; future research may further reveal the complex mechanisms of the joint effects of the semantic and structural dimensions by introducing more comprehensive engagement indicators and dynamic network analysis methods.

Second, all findings of this study represent cross-sectional associations and do not constitute grounds for causal inference. The propensity score matching supplementary analysis maintained a directionally consistent propagation difference between within-level and cross-level combinations after controlling for network centrality and co-occurrence frequency, but the effect size was extremely small, suggesting that part of the propagation difference may be realized through the network centrality pathway rather than being produced by cognitive compatibility alone. The possibility of reverse causality cannot be fully ruled out: vernacular combinations in popular posts may be imitated by subsequent users, thereby reinforcing the observed association patterns through selection effects. Potential omitted variables, including poster follower count, historical engagement baseline, posting time, and ACG content subcategory, were not controlled for and may constitute sources of confounding. Additionally, the NER classifier’s validation is based on an internal test set and has not been conducted on an independent external corpus, which limits the assessment of the classification scheme’s cross-platform transferability; internal robustness has been addressed through per-class performance evaluation and reclassification noise simulation, and future research should conduct external validation on other platform corpora.

Third, platform community vernacular systems are dynamically evolving structures; both the cognitive hierarchy attribution and network structural position of color vernaculars may shift as subcultural developments unfold, and the cross-sectional design of this study cannot capture this dynamic process. Therefore, replicating the core findings in other platforms and content types with stratified community vernacular attributes, and introducing longitudinal data to test the temporal stability and cross-contextual generalizability of the dual-dimensional association patterns, are worthwhile directions for future research.

## Conclusion

This study evaluated the association patterns of semantic hierarchy compatibility and network structural bridging on the propagation outcomes of community vernacular combinations in social media platform contexts, and further examined the functional form of the cognitive distance effect, the propagation associations of network centrality embedding positions, and the moderating pattern of cognitive compatibility on structural bridging effects, while analyzing the theoretical implications of the dual-dimensional framework for understanding platform content propagation mechanisms. The following primary conclusions are drawn.

First, a systematic positive association exists between semantic hierarchy compatibility and combination propagation synergy effects; within-level cognitively compatible combinations correspond to higher propagation gains, cross-level cognitively incompatible combinations correspond to negative synergy effects, and this association remains consistent across robustness subset analyses. Second, the association between cognitive distance and propagation outcomes follows a monotonically accelerating declining pattern; the quadratic term is significantly negative while the linear term is non-significant, and the inverted-U optimal interval hypothesis is not supported in the social media context of this study, with the findings more consistent with the literature lineage of monotone negative relationships. Third, the direction of association between network centrality and propagation outcomes changes systematically with sample frequency thresholds, showing a positive association among high-frequency stable node pairs and a negative association among low-frequency full-sample node pairs, indicating systematic differences in the propagation implications of centrality across node pairs with different frequency distributions. Fourth, semantic hierarchy and community relationship associate with propagation outcomes in opposite directions; cross-level semantics correspond to a negative association and structural cross-community spanning corresponds to a positive association, and cross-community combinations with higher cognitive hierarchy compatibility show significantly higher mean propagation outcomes than cross-community combinations with cognitive hierarchy incompatibility, forming an identifiable asymmetric dual-dimensional association pattern.

The findings of this study, in the context of the Xiaohongshu ACG color vernacular network, indicate that platform semantic symbol propagation outcomes are simultaneously associated with both the cognitive-semantic dimension and the network structural dimension, the two dimensions associate in opposite directions and are not interchangeable, and they must be understood within a dual-dimensional integration framework. Future research needs to continue advancing cross-contextual comparative verification in other platform contexts and other types of community vernacular systems, and to introduce longitudinal data to test the temporal stability and degree of generalizability of the above association patterns.

## Supplementary Information

Below is the link to the electronic supplementary material.

Supplementary Material 1

## Data Availability

The datasets generated and analyzed for this study can be found in the Zenoda repository at http://doi.org/10.5281/zenodo.17473792.
