# Supplementary material for: Asymmetric effects of semantic compatibility and structural bridging on content propagation: evidence from community vernacular combinations on social media platforms
Source: Sci Rep. 2026 May 9;16:21230. doi: 10.1038/s41598-026-51438-6 (PMC13346501; doi:10.1038/s41598-026-51438-6)
Supplement: Supplementary file 1 — Supplementary Material 1 [file 41598_2026_51438_MOESM1_ESM.docx]

## Supplementary Materials

## Supplementary Table S1. Instance-level OLS with Pair-Clustered Standard Errors

| **Model** | **n (instances)** | **n (pairs)** | **β (cross-level, binary)** | **SE** | **t** | **p** | **95% CI** | **Adj R²** |
| --- | --- | --- | --- | --- | --- | --- | --- | --- |
| Instance-level OLS + pair-clustered SE | 55,890 | 21,105 | −0.254 | 0.015 | −17.163 | <0.001 | [−0.283, −0.225] | 0.053 |
| Instance-level OLS + HC3 (reference) | 55,890 | 21,105 | −0.254 | 0.013 | −19.923 | <0.001 | [−0.279, −0.229] | 0.053 |
| Pair-level OLS + HC3 (primary analysis) | 21,105 | 21,105 | −0.284 | 0.015 | −18.517 | <0.001 | [−0.314, −0.254] | 0.030 |

Note: *All models include log(n_instances) as a control variable (standardized). Pair-clustered SE accounts for non-independence of multiple posts contributing to the same color pair. The pair-clustered SE is slightly larger than HC3 (0.0148 vs. 0.0127), confirming that clustering yields more conservative inference. Coefficients across all three models are directionally consistent, confirming that pair-level aggregation does not introduce inferential bias.*

## Supplementary Table S2. Propensity Score Matching Results

**S2-A. Propensity Score Logit Model Coefficients**

| **Parameter** | **beta** | **SE** | **z** | **p** | **95% CI lower** | **95% CI upper** | **Pseudo R2** |
| --- | --- | --- | --- | --- | --- | --- | --- |
| Intercept | 0.2571 | 0.0185 | 13.884 | <0.001 | 0.2208 | 0.2933 | 0.313 |
| log_n_z | -0.8084 | 0.0237 | -34.122 | <0.001 | -0.8548 | -0.7620 | 0.313 |
| avg_degree_z | 4.1521 | 0.1158 | 35.864 | <0.001 | 3.9251 | 4.3790 | 0.313 |
| avg_pr_z | -2.4879 | 0.1141 | -21.809 | <0.001 | -2.7115 | -2.2643 | 0.313 |

**S2-B. Covariate Balance (Standardized Mean Difference)**

| **Covariate** | **SMD Before Matching** | **SMD After Matching** | **Balance (< 0.1)** |
| --- | --- | --- | --- |
| log_n | 0.1129 | 0.0006 | Yes |
| pair_avg_degree | 1.3134 | 0.0449 | Yes |
| pair_avg_pagerank | 1.2210 | 0.0747 | Yes |
| Propensity score | 1.6247 | 0.0002 | Yes |

**S2-C. Matched-Pair Effect Estimate**

| **Statistic** | **Value** |
| --- | --- |
| Full sample within-level n | 10,051 |
| Full sample cross-level n | 11,054 |
| Common support region n | 19,702 |
| Matched pairs | 3,813 |
| Matching rate (%) | 38.6% |
| Matched within-level mean | 0.7679 |
| Matched cross-level mean | 0.7244 |
| Mean difference (within - cross) | 0.0436 |
| Difference SE | 0.0217 |
| 95% CI of difference | [0.0011, 0.0860] |
| Paired t statistic | 2.0128 |
| p value | 0.0442 |
| Cohen's d (paired) | 0.0326 |
| Test method | Paired t-test (1:1 matched-pair differences) |

**S2-D. Unmatched Cross-Level Pairs: Descriptive Comparison**

| **Group** | **n** | **Mean pair_avg_degree** | **Mean log_n** | **Mean propagation** |
| --- | --- | --- | --- | --- |
| All cross-level | 9,875 | 0.1197 | 0.7961 | 0.5973 |
| Matched subset (cross-level) | 3,813 | 0.0709 | 0.8523 | 0.7244 |
| Unmatched (cross-level) | 6,062 | 0.1504 | 0.7607 | 0.5174 |

*Note: Double-caliper matching achieves balance on all four covariates (SMD < 0.1 post-matching). The matched within-level mean (0.768) exceeds the matched cross-level mean (0.724); paired t = 2.013, p = 0.044, Cohen's d = 0.033 (very small effect). Unmatched cross-level pairs show higher avg_degree and lower propagation than matched pairs, indicating that the unmatched group disproportionately represents high-centrality, lower-propagation pairs. The very small effect size after matching suggests that part of the raw propagation difference is realized through the network centrality pathway.*

## Supplementary Table S3. NER Reclassification Noise Simulation

**Supplementary Table S3-A. Cross-level Classification Coefficient Robustness**

| **Noise rate** | **Mean β** | **SD β** | **β range** | **OLS significance rate (%)** | **Directional accuracy (%)** |
| --- | --- | --- | --- | --- | --- |
| 0% | −0.284 | 0.000 | [−0.284, −0.284] | 100.0 | 100.0 |
| 2% | −0.272 | 0.004 | [−0.285, −0.262] | 100.0 | 100.0 |
| 5% | −0.255 | 0.006 | [−0.271, −0.245] | 100.0 | 100.0 |
| 10% | −0.227 | 0.009 | [−0.245, −0.212] | 100.0 | 100.0 |

*Note: "Noise rate" refers to the proportion of cross-level classification labels randomly flipped. Significance rate: proportion of 50 repetitions in which β (cross-level, binary) achieves p < 0.05. Directional accuracy: proportion in which β is negative (correct direction).*

**Supplementary Table S3-B. Cognitive Distance Effect Robustness**

| **Noise rate** | **Mean JT z** | **SD JT z** | **JT monotone rate (%)** | **Mean quadratic β** | **SD quadratic β** | **Quadratic neg. sig. rate (%)** |
| --- | --- | --- | --- | --- | --- | --- |
| 0% | 17.448 | 0.000 | 100.0 | −0.061 | 0.000 | 100.0 |
| 2% | 17.182 | 0.531 | 100.0 | −0.051 | 0.003 | 100.0 |
| 5% | 16.645 | 0.680 | 100.0 | −0.039 | 0.004 | 100.0 |
| 10% | 15.725 | 0.685 | 100.0 | −0.025 | 0.005 | 100.0 |

*Note: "Noise rate" refers to the proportion of cognitive distance values randomly shifted by ±1. Monotone rate: proportion in which group means strictly decrease across cognitive distance levels 0, 1, and 2. Quadratic neg. sig. rate: proportion in which the quadratic term is negative and p < 0.05.*

## Supplementary Table S4. Symbolic-Level Exclusion Sensitivity Analysis

**S4-A. Main Tests: Full Propagation Outcome and Synergy Effect Samples**

|  | **Including Symbolic (full)** | **Excluding Symbolic** |
| --- | --- | --- |
| Total pairs | 21,105 | 19,887 |
| Within-level n | 10,051 | 9,780 |
| Cross-level n | 11,054 | 10,107 |
| Within-level mean propagation | 0.8611 | 0.8886 |
| Cross-level mean propagation | 0.5939 | 0.6481 |
| Propagation difference (within - cross) | 0.2673 | 0.2405 |
| Welch t | 17.307 | 14.993 |
| p | <0.001 | <0.001 |
| Cohen's d | 0.238 | 0.212 |
| OLS beta (cross-level) | -0.284 | -0.260 |
| OLS SE | 0.015 | 0.016 |
| OLS 95% CI | [-0.314, -0.254] | [-0.291, -0.228] |
| Synergy pairs | 3,108 | 3,050 |
| Mean synergy | 0.398 | 0.408 |
| Synergy overall t | 22.527 | 22.735 |
| Within-level synergy mean | 0.461 | 0.461 |
| Cross-level synergy mean | 0.330 | 0.348 |
| Synergy Welch t | 3.706 | 3.133 |
| Synergy p | <0.001 | 0.002 |

**S4-B. Cognitive Distance Group Decomposition**

| **Dataset** | **d** | **n** | **Mean** | **SD** | **95% CI lower** | **95% CI upper** |
| --- | --- | --- | --- | --- | --- | --- |
| Including symbolic | 0 | 10,051 | 0.8611 | 1.0929 | 0.840 | 0.883 |
| Including symbolic | 1 | 10,364 | 0.6336 | 1.1650 | 0.611 | 0.656 |
| Including symbolic | 2 | 690 | -0.0037 | 0.6485 | -0.052 | 0.045 |
| Excluding symbolic | 0 | 9,780 | 0.8886 | 1.0930 | 0.867 | 0.910 |
| Excluding symbolic | 1 | 10,107 | 0.6481 | 1.1691 | 0.625 | 0.671 |
| Excluding symbolic | 2 | 0 | -- | -- | -- | -- |

*Note: After excluding symbolic-level pairs, the directional pattern of all primary conclusions is preserved: the cross-level coefficient remains negative and significant, the within-level synergy advantage remains significant (p = 0.002), and the monotone decline between d = 0 and d = 1 remains intact (0.889 vs. 0.648, p < 0.001). The d = 2 group becomes empty after exclusion because it is structurally composed entirely of symbolic-level pairs, confirming that the d = 2 result in the primary analysis reflects the presence of symbolic-level vocabulary rather than a spurious artifact.*
